# Animal and Vegetable Parasites of the Human Skin and Hair

**Published:** 1872-05

**Authors:** 


*Animal and Vegetable Parasites of the Human Skin and Hair.* By B. JOY JEFFRIES, A. M., M. D. etc. Boston: ALEXANDER MOORE, 1872.

The person who seeks in this little book a full and complete treatise on Animal and Vegetable Parasites of the human body will be compelled to turn away disappointed, but he who seeks a work on this subject suitable for the popular reader, and suggestive and instructive to the physician will find his want supplied.

The author speaks in turn of the different kinds of parasites and gives plain and concise directions as to the steps to be taken to get rid of these troublesome pests.

